# Factors influencing the social participation ability of rural older adults in China: A cross-sectional study

**DOI:** 10.3389/fpubh.2022.1001948

**Published:** 2023-01-04

**Authors:** Minghui Cheng, Wenli Su, Huiju Li, Linjie Li, Minghui Xu, Xue Zhao, Mengdie Han, Li Yang

**Affiliations:** ^1^School of Nursing, Lanzhou University, Lanzhou, Gansu, China; ^2^Fenyang College of Shanxi Medical University, Fenyang, Shanxi, China; ^3^Internal Department of Special Care, Lanzhou University Second Hospital, Lanzhou, Gansu, China

**Keywords:** elder adults, social participation, level, influencing factors, cross-sectional

## Abstract

**Objective:**

To investigate the epidemiology and influencing factors of social participation ability of rural older adults in China.

**Methods:**

From March to April 2021, 3450 older adults in poverty aged 60 and above registered in Jishishan County (J County) were selected by cluster sampling for a cross-sectional questionnaire survey and their social participation ability was assessed using the Ability Assessment of older adults (MZ/T039-2013). The results were statistically analyzed and an ordered multi-category logistic regression analysis was used to analyze the effect of influencing factors on the social participation ability of rural older adults.

**Results:**

3,346 questionnaires were collected, with an effective recovery rate of 96.99%. Out of all the participants, 1,355 (40.5%) of the 3,346 cases had intact social participation ability, while 1,991 (59.5%) had different degrees of loss of social participation ability, of which 1,393 (41.14%) were mildly impaired, 419 (12.5%) were moderately impaired and 179 (5.3%) were severely impaired. Age, educational level, religious belief, living status, whether suffering from dementia and the occurrence of accidents in recent 30 days were influencing factors on the social participation ability (*p* < 0.05).

**Conclusion:**

The rate of impaired social participation ability among older adults was >50% and age, educational level, religious beliefs, living status, whether suffering from dementia, and the occurrence of accidents in recent 30 days (such as falls, choking, loss) were significant factors influencing the ability of social participation of rural older adults.

## 1. Introduction

The promotion of active aging has become a common practice adopted by the international community to combat the challenges associated with an aging population. One of the core elements of active aging is the social participation of older adults (1–4). Social participation is a form of social interaction or community involvement that may include activities with friends, family, and other individuals, telephone conversations, religious activities, participation in cultural activities, or participation in exercise groups (5). In addition to the societal benefits of social participation by the elderly (6), accumulating evidence has shown that social participation can help to reduce the costs of long-term elderly care and help to improve individual psychological and health conditions. Indeed, participation in social activities has been demonstrated to positively impact rates of depression and loneliness, and to improve cognitive functioning (5–8).

Previous studies (9, 10) have identified age, level of education, marital status, and health conditions as influencing factors of social participation. However, these studies have focused on urban older adults or patients with identified diseases while little is known about social participation and the influencing factors thereof among rural older adults in impoverished areas. Jishishan County (J County) has unique characteristics (11), as the only multi-ethnic autonomous county in Gansu Province. At the same time, it is a poverty-stricken county, and its economic and social development is far behind that of Gansu Province and of the rest of the country. This is also reflected in material scarcity and in a lack of access to tangible and intangible resources (11). To this end, social participation, a process that facilitates information exchange, strengthens ties to the community and access to resources, promotes a healthy physiological state, and fosters social support systems, may bring social and economic upliftment to J country, particularly to its elderly inhabitants.

Therefore, this study sought to investigate the capacity for social participation of elderly inhabitants in the J county region of rural China and the factors influencing this social participation in the aim of promoting active aging.

## 2. Materials and methods

### 2.1. Study design and participants

A cross-sectional study was conducted in the rural J county of Gansu province in northwest China from March to April 2021. Participants were recruited using a cluster sampling method. The inclusion criteria for participants were set as (1) aged >60 years old and (2) voluntary participation in the survey. If the participants are unable to communicate directly with investigators due to mental, speech, or hearing impairments, the family members were allowed to answer the questions. Exclusion criteria: (1) the death of a registered older adult and/or (2) the individual was no longer in the locality or was uncontactable during the course of the study.

### 2.2. Procedures

Data was collected through means of a questionnaire. The cover page of the questionnaire explained the research purpose and process. Participants signed an informed consent form before filling out the questionnaire. The questionnaires were filled out in private with investigators on-site to handle any questions. The completeness of the questionnaires was checked on-site by the investigators. Three thousand and four hundred and fifty older adults were invited to participate, and 104 incomplete questionnaires were excluded. Ultimately, 3,346 (96.99%) valid questionnaires were analyzed and included in this study.

### 2.3. Measurements

The “Ability Assessment of the older adults” (MZ/T039-2013) was used to evaluate the respondents. This assessment tool was developed by the Ministry of Civil Affairs of the People's Republic of China. It has good reliability and validity, with a Cronbach's α score of 0.889. The assessment contains two parts: (1) Basic information about the older adults, including gender, age, education level, religion, marital status, living status, and accidents in the recent 30 days (e.g., choking, falls, or having gone missing). (2) The social participation ability assessment section of the form included five items of life ability, ability to work, time/space orientation, person orientation, and social interaction ability. Each item was scored by participants on a 5-point scale, ranging from 0 to 4, with a total possible score of 20 points. The social participation ability was assessed according to a score of 0 to 2. A total score of 3–7 was categorized as level 1, indicating mild impairment; a total score of 8–13 was assigned to level 2, indicating moderate impairment; a total score of 14–20 was assigned to level 3, indicating a severe impairment.

### 2.4. Statistical methods

After the questionnaires were collected, the data were initially organized using Excel, and SPSS 26.0 statistical software was used for statistical analysis. The (M ± SD) was used to assess the data, and the counted data were described by frequency and composition ratio. Nonparametric statistical tests (Mann-Whitney U test and Kruskal-Wallis test) were used for Univariate analysis and ordered multi-categorical logistic regression analyses were used to explore the effect of the influencing factors on the level of social participation. A difference where *P* < 0.05 was regarded as statistically significant.

### 2.5. Ethical considerations

Before enrolling in the study, eligible participants signed a consent form. After the survey was completed, all data were stored anonymously without names or identifying information to protect participants' confidentiality.

## 3. Results

### 3.1. Basic characteristics of rural older adults

A total of 3,346 older adults were collected in this study, with an effective recovery rate of 96.99%, of which 48.8% were male. In terms of the age of participants, 1,278 cases (38.2%) were aged 60 and above, 1,529 cases (45.7%) were aged 70 and above, and 539 cases (16.1%) were aged 80 and above. Most of the older adults (88.5%) were illiterate (Table 1).

**Table 1:** Demographic characteristics and univariate analysis of social participation.

	**Intact**	**Mild**	**Moderate**	**Severe**	**Total**	***Z/H***	***P***
**Gender**							
Male	685(41.9%)	693(42.4%)	181(11.1%)	74(4.5%)	1,633	−2.701	0.007
Female	670(39.1%)	700(40.9%)	238(13.9%)	105(6.1%)	1,713		
**Age(years)**							
60~	671(52.5%)	474(37.1%)	94(7.4%)	39(3.1%)	1,278	268.089	0.000
70~	590(38.6%)	672(44.0%)	199(13.0%)	68(4.4%)	1,529		
80~	94(17.4%)	247(45.8%)	126(23.4%)	72(13.4%)	539		
**Nationality**							
Han	566(40.7%)	576(41.4%)	166(11.9%)	82(5.9%)	1,390	23.528	0.001
Hui	579(40.1%)	618(42.8%)	178(12.3%)	68(4.7%)	1,443		
Dongxiang	72(30.3%)	106(44.5%)	42(17.6%)	18(7.6%)	238		
Baoan	89(49.4%)	57(31.7%)	25(13.9%)	9(5.0%)	180		
Sala	35(51.5%)	25(36.8%)	6(8.8%)	2(2.95)	68		
Tu	10(47.6%)	9(42.9%)	2(9.5%)	0(0.0%)	21		
Zang	4(66.7%)	2(33.3%)	0(0.0%)	0(0.0%)	6		
**Educational level**							
illiteracy	1,137(38.4%)	1,263(42.7%)	390(13.2%)	170(5.7%)	2,960	−7.029	0.000
literacy	218(56.5%)	130(33.7%)	29(7.5%)	9(2.3%)	386		
**Religion**							
Without	534(43.3%)	493(40.0%)	135(10.9%)	71(5.8%)	1,233	−2.367	0.018
With	821(38.9%)	900(42.6%)	284(13.4%)	108(5.1%)	2,113		
**Marital status**							
Single/divorced/widowed	396(29.2%)	474(34.0%)	155(37.0%)	97(54.2%)	1,122	−5.638	0.000
Married	959(70.8%)	919(66.0%)	264(63.0%)	82(45.8%)	2,224		
**Living status**							
live alone	129(45.7%)	108(38.3%)	30(10.6%)	15(5.3%)	282	141.586	0.000
Live with spouse	885(46.9%)	761(40.3%)	185(9.8%)	55(2.9%)	1,886		
Live with others	341 (35.3%)	524(44.5%)	204(17.3%)	109(9.3%)	1,178		
**Dementia**							
Without	1,346(41.2%)	1,366(41.8%)	401(12.3%)	152(4.7%)	3,265	−8.432	0.000
With	9(11.1%)	27(33.3%)	18(22.2%)	27(33.3%)	81		
**Number of chronic diseases**							
None	593(42.5%)	539(38.6%)	178(12.8%)	86(6.2%)	1,396	4.619	0.099
1~2	712(39.7%)	781(43.5%)	217(12.1%)	84(4.7%)	1,794		
3~	50(32.1%)	73(46.8%)	24(15.4%)	9(5.8%)	156		
**Accidents in recent 30d**							
Without	1,208(42.4%)	1,153(40.5%)	352(12.4%)	136(4.8%)	2,849	−5.355	0.000
With	147(29.6%)	240(48.3%)	67(13.5%)	43(8.7%)	497		

### 3.2. Rating of social participation ability of older adults

The results showed that 558 (16.7%) had complete life skills, 98 (2.9%) had complete work skills, 1,950 (58.3%) had complete time/space orientation skills, 2,598 (77.6%) had complete person orientation skills, and 2,343 (70.0%) had complete social interaction skills. Moreover, the results showed that 1,355 (40.5%) had intact social participation ability; 1,991 had varying degrees of impaired social participation ability, with an impairment rate of 59.5%, of which the mild, moderate, and severe damage rates were 41.14% (1,393/3,346), 12.5% (156/3,346) and 5.3% (62/3,346), respectively.

### 3.3. Univariate analysis of factors associated with social participation ability

The analysis showed that gender, age, ethnicity, education, religious belief, marital status, living status, whether suffering from dementia and the occurrence of accidents in the past 30 days were factors influencing the ability to participate in social interactions (*p* < 0.05, Table 1).

### 3.4. Ordered multi-categorical logistic regression analysis of the influencing factors of social participation

An ordered multi-categorical logistic regression analysis was conducted using the social participation ability level of the older adults as the dependent variable (intact = 0, mildly impaired = 1, moderately impaired = 2, and severely impaired = 3), and gender, age, ethnicity educational level, religious belief, marital status, residence status, diagnosis of dementia, and accident incidence in the past 30 days as the independent variables. There was no multicollinearity found among the independent variables (VIF in the range of 1.005 to 4.688) and the parallelism test met the requirements (χ^2^ = 41.804, *P* = 0.115). In summary, the results showed that age, educational level, religion, marital status, living status, and whether participants had dementia, and/or accidents in the past 30 days were influential factors in the social participation ability of the rural older adults (*P* < 0.05, Table 2).

**Table 2:** Ordered multi-categorical logistic regression analysis of influencing factors of social participation level.

	**B**	**SE**	***Wald χ^2^***	***P***	***OR (95%CI)***
**Gender**					
Male	−0.015	0.070	0.049	0.825	0.99 (0.14–5.62)
**Age (years)**					
60~	−1.479	0.103	204.215	0.000	0.23 (0.19–0.28)
70~	−0.969	0.097	100.181	0.000	0.38 (0.31–0.46)
**Nationality**					
Han	0.998	0.901	1.229	0.268	2.71 (0.46–15.85)
Hui	0.233	0.904	0.066	0.797	1.26 (0.21–7.42)
Dongxiang	0.838	0.910	0.847	0.357	2.31 (0.39–13.76)
Baoan	−0.001	0.914	0.000	0.999	0.80 (0.13–5.02)
Sala	−0.217	0.934	0.054	0.816	1.03 (0.16–6.80)
Tu	0.487	1.001	0.237	0.626	1.63 (0.23–11.58)
**Educational level**					
illiteracy	0.665	0.115	33.409	0.000	1.94 (1.55–2.44)
**Religion**					
Without	−0.801	0.144	30.890	0.000	0.45 (0.34–0.60)
**Marital status**					
Single/divorced/ widowed	−0.559	0.117	22.847	0.000	0.57 (0.45–0.72)
**Living status**					
Live alone	−0.542	0.131	17.030	0.000	0.58 (0.45–0.75)
Live with spouse	−1.017	0.109	87.001	0.000	0.36 (0.29–0.45)
**Dementia**					
Without	−1.987	0.212	87.869	0.000	0.14 (0.09–0.21)
**Accidents in recent 30d**					
Without	−0.514	0.093	30.624	0.000	0.60 (0.50–1.39)

## 4. Discussion

### 4.1. High rate of impaired social participation among rural older adults

This study used the “Ability Assessment of the older adults” to determine the social participation ability of older adults with time/space orientation, person orientation, and other dimensions taken into account to diversify the measurements of social participation level of older adults (12). In this study, the social participation ability of older adults was intact in 1,355 cases, accounting for 40.5%, and impaired to varying degrees in 1,991 cases, with an impairment rate of 59.5% which was much higher than the results of a study by Shi et al. (12) on the level of social participation of rural elderly in Henan province (20.84%).

The social participation ability of the rural older adults was found to be inconsistent with the literature in several dimensions. Among them, the proportion of intact character orientation ability was 77.6%, which was inconsistent with previous research results (where the proportion was 92.78%) (12). Similarly, the proportion of intact ability in the five dimensions of life ability (16.7%), workability (2.9%), time/space orientation ability (58.3%), person orientation ability (77.6%), and social interaction ability (70.0%) were all lower than that observed in previous studies (12). The reasons for these differences may include: (1), appropriate income as an important contributor to achieving active participation of older adults in society (13). Indeed, J County was, until recently, one of the 23 counties in the province with deepest levels of poverty and although it exited poverty status in 2020 the economic level is still considerably low compared to other provinces and cities, especially for poor older adults. Consequently, older adult residents are under more significant pressure to meet their basic needs to survive. Secondly, the high number of ravines in the mountainous areas of County J and the lack of access to transportation means that older people have fewer interactions with the outside world; most of them live in mountainous areas, with long distances between families and less communication between neighbors. Thirdly, the low average level of education of the population in the area (with an illiteracy rate of 88.5%) and the limited knowledge and skill levels of the population in the use of modern communication and other forms of media limits the content and methods of social participation available to this population.

### 4.2. Factors influencing the social participation of older adults

In this study, the risk of impaired social participation ability was 0.23 and 0.38 times higher among 60–69 and 70–79-year-olds respectively than among 80-year-olds and above. Guo et al. (14) showed that after controlling the effects of other variables, the level of social participation ability among older urban adults in Guangdong Province was significantly higher in the lower age group (60–75 years) than in the higher age group (>75 years), which is consistent with our results. In addition, aging inevitably leads to increased incidence of various physical ailments (15), making older people's social participation more homogeneous and narrowed in terms of the range of social participation activities. This leads to an increased rate of impaired social participation in this group, in line with previous research findings (16). Therefore, older adults should be encouraged to participate in various activities to improve their social participation ability according to their health status and physical and cognitive abilities.

The results of this study showed that illiterate older adults are 1.94 times more at risk of impaired social participation than literates, which is consistent with the results of existing studies (15, 16). The higher the level of education and the more open-minded and receptive people are to new things, the relatively more motivated people are to participate in society (17) the easier it is to integrate into the society's culture. However, Liu et al. and Yang et al. (18, 19) found a low correlation between the educational level and social participation of older people. This difference may be due to the small number cohort of the two studies or due to the use of the different research methods. Our findings indicate that we should pay more attention to the lesser-educated older adults and use the village council as a vehicle to change their attitudes toward social participation, using methods such as publicity and peer education to encourage active participation in social activities.

The results of this study showed that dementia and unexpected life events (falls, suicide, being lost) within the last 30 days were influential factors on social participation ability. Yang et al. (20) proposed that chronic diseases and mental illness constrain the social participation of older people in China. Due to the absence or abnormality of one or more physical or mental functions, older adults with such diseases have a weaker ability to take care of themselves (21). The reduced ability of self-care not only limits their ability to be socially active but also leads to adverse emotional reactions in older adults, thus potentially limiting their ability to engage in social participation (22). Older adults with functional disabilities should therefore be provided with appreciative social activities based on maintaining their abilities.

Compared to older adults without spouses, those with spouses are more likely to participate in social activities. Shi et al. (12) conducted a questionnaire survey on rural older adults aged 60 and above in Henan Province, and the results showed that the social participation ability of the older adults with spouses was higher than that of the older adults without spouses, which was consistent with the results of this study. In China, where kinship is vital, living with a spouse or relative enhances the level of family support for older people, which can significantly increase their sense of well-being (23), and improves their ability to participate in society. For older adults living alone, the lack of family companionship can cause loneliness in their lives and can be detrimental to their psychological well-being, leading to a decline in their social participation ability. Therefore, older adults living alone should be encouraged to participate in social activities when their physical condition allows, and at the same time, it is recommended that more social services and financial support be provided to those living with other relatives.

### 4.3. Strengths and limitations

This study is the first large-scale survey on the social participation ability of older adults in poor areas in China. Our findings may help to improve the capacity of the government, village committees, social workers, and other social forces in helping to strengthen social participation and build social support systems for older adults in poor areas. There are limitations to this study: (1) Due to the survey data, this study only analyses some aspects of social participation and does not study the specific content of social participation. This study could be improved in future research, by collecting more comprehensive data on social participation, and by exploring the relevant influencing factors in depth. (2) The study investigated the social participation ability of older adults in J County. As J County is a gathering place of ethnic minorities and its economic level is of a low-income society, the results of the study may not represent the social participation level of older adults in the country. (3) The data is all self-reported and is therefore based on participants perceived ability rather than their tested ability.

## 5. Conclusion

In conclusion, the rate of impaired social participation of rural older adults in J county, China is 59.5%, and age, religion, education level, living status, and functional deficits are important influencing factors of social participation ability. Ensuring the integrity of older rural adults' social participation ability can effectively reduce the medical care burden for older adults in rural areas, where social participation can significantly promote healthy aging in China. In order to improve the social participation ability of rural older adults, firstly the township hospital needs to be equipped with professional medical staff to monitor the health of older adults, regularly promote health information, and provide question-answering and consulting services. Secondly, given that older adults in J County are currently largely limited to a single form of recreation and a few amateur cultural activities, it is necessary to speed up the promotion of public services for recreation and culture, reduce the difference between urban and rural areas and promote the participation of older adults in social activities in rural areas. We can strengthen rural infrastructure through financial subsidies, social donations, and corporate philanthropy to provide a concrete material basis for the social participation of older people in rural areas. Finally, the low level of social participation of older people in J county may be because older people have fewer channels to obtain information about these activities. We should therefore use appropriate tools to publicize the activities that are available to older adults broaden the publicity channels, and promote social participation overall.

## Data availability statement

The raw data supporting the conclusions of this article will be made available by the authors, without undue reservation.

## Author contributions

MC, WS, HL, and LY contributed to conception and design of the study. LL, MX, XZ, and MH wrote sections of the manuscript. All authors contributed to the article and approved the submitted version.
